# There is no shortage, but inequality: demographic evolution of neurologists in Brazil (2010–2020)

**DOI:** 10.1055/s-0043-1761490

**Published:** 2023-03-22

**Authors:** Bruno Lopes Santos-Lobato, Pedro José Tomaselli, Edienny Augusta Viana Santos-Lobato, Alex Jones Flores Cassenote, Hideraldo Luis Souza Cabeça

**Affiliations:** 1 Universidade Federal do Pará, Laboratório de Neuropatologia Experimental, Belém PA, Brazil.; 2 Hospital Ophir Loyola, Belém PA, Brazil.; 3 Universidade de São Paulo, Faculdade de Medicina de Ribeirão Preto, Departamento de Neurociências e Ciências do Comportamento, Ribeirão Preto SP, Brazil.; 4 Universidade do Estado do Pará, Programa de Pós-Graduação de Educação em Saúde na Amazônia, Belém PA, Brazil.; 5 Universidade de São Paulo, Faculdade de Medicina de São Paulo, São Paulo SP, Brazil.

**Keywords:** Neurology, Brazil, Demography, Socioeconomic Factors, Neurologia, Brasil, Demografia, Fatores Socioeconômicos

## Abstract

**Background** Neurology is a medical specialty that deals with prevalent diseases such as stroke, headache, epilepsy, and neurodegenerative diseases. Many countries, such as Brazil, struggle to provide neurological care for their populations, but the inadequacy and unequal distribution of the neurologist workforce are real challenges.

**Objective** To analyze the demographic evolution of neurologists and the first-year Neurology residency positions in Brazil during the last decade (2010–2020) and the distribution imbalance between regions.

**Methods** The demographic and geographic distribution of neurologists was calculated based on data extracted from the Brazilian Federal Medical Council reports, and the number of Neurology residency positions was based on the Brazilian National Commission of Medical Residency reports. Indicators of wealth were associated with demographic data.

**Results** The number of neurologists per 100,000 population has increased since 2011, with a similar increase in the geographic distribution of neurologists. However, there was a marked inequality of distribution of neurologists through regions, with a gap between the Northern (lowest) and Southeastern (highest) regions. Furthermore, the imbalance of distribution of neurologists strongly correlated with social inequality. The number of Neurology residency positions increased, but with an imbalance between North and Southeast regions.

**Conclusions** Brazil has advanced in providing neurologists. However, instead of a shortage, inequality between regions is the greatest challenge regarding the neurological workforce. The training of new neurologists is unequal between regions and occurs at a slower rate than needed. Neurologists, public health authorities, and patients should discuss solutions for these issues.

## Resumo

**Antecedentes** A Neurologia é uma especialidade médica que lida com doenças prevalentes, como acidente vascular cerebral, cefaleia, epilepsia e doenças neurodegenerativas. Muitos países, como o Brasil, se esforçam para oferecer assistência neurológica à população, mas a distribuição insuficiente e desigual da força de trabalho de neurologistas são desafios.

**Objetivo** Analisar a evolução demográfica dos médicos neurologistas e das vagas de Programas de Residência Médica em Neurologia no Brasil durante a última década (2010-2020) e o desequilíbrio de distribuição entre as regiões.

**Métodos** A distribuição demográfica e geográfica de neurologistas foi calculada com base nos dados extraídos de relatórios do Conselho Federal do Medicina do Brasil, e o número de vagas em Programas de Residência Médica em Neurologia foi extraído de dados da Comissão Nacional de Residência Médica. Os indicadores de riqueza foram associados aos dados demográficos.

**Resultados** O número de neurologistas por 100.000 habitantes aumentou desde 2011, com um aumento similar na distribuição geográfica de neurologistas. Entretanto, houve uma nítida desigualdade na distribuição de neurologistas entre as regiões, com um hiato entre as regiões Norte e a Sudeste. Além disso, a desigualdade da distribuição de neurologistas se correlacionou fortemente com a desigualdade social. O número de vagas em Programas de Residência Médica aumentou, porém com desigualdade entre as regiões Norte e Sudeste.

**Conclusões** O Brasil tem avançado na geração de neurologistas. Porém, ao invés de uma escassez, a desigualdade entre regiões é o maior desafio em relação à força de trabalho neurológica. O treino de novos neurologistas é desigual entre regiões e ocorre em um ritmo mais lento do que o necessário. Neurologistas, autoridades em saúde pública e pacientes devem discutir soluções para estes problemas.

## INTRODUCTION

Neurology is a medical specialty that is constantly changing and evolving. In recent decades, the global incidence and prevalence of some neurological diseases, like stroke, Alzheimer, and Parkinson disease, has dramatically increased. 1 Additionally, a substantial proportion of patients may develop neurological complications due to the COVID-19 pandemic. 2 Therefore, it is clear that there is a need for a considerable contingent of well-trained neurologists to meet the population's demands.

The United Kingdom and the World Health Organization (WHO) recommended a minimum of one neurologist per 100,000 population, based on a report written in 1997 on behalf of the Association of British Neurologists. 3 Nowadays, low-, middle-, and high-income countries struggle to increase their Neurology workforce. In the USA, the supply of neurologists may increase by 11% between 2013 and 2025, but the demand for neurological care may grow by 16%. 4 Neurologists are unevenly distributed in some countries, with a reduced Neurology workforce mainly in less attractive regions and rural areas. 5

Brazil also has problems with its Neurology workforce, 6 7 8 primarily due to regional imbalance, instead of low absolute numbers of neurologists. For example, the Brazilian Amazon (Northern region) has been the region with fewer neurologists. This scarcity aggravates its fragile health infrastructure and unique geography, marked by the Amazon rainforest and numerous rivers. 8

Since 2011, the Brazilian Federal Medical Council has published a thorough report on the Brazilian medical demography for the years 2011, 2013, 2015, 2018, and 2020. 9 10 11 12 13 The data may shed light on the evolution of the number of neurologists in Brazil and their distribution through regions in the last decade and, together with new data on the numbers of Neurology residency programs, a more precise diagnosis about the adequacy of the neurologist workforce in Brazil can be made. This study aimed to analyze the evolution of numbers of neurologists and first-year Neurology residency positions in Brazil during the last decade (2010–2020) and their distribution through regions and states. We will also explore indicators of social inequality in the distribution of neurologists throughout the country.

## METHODS

### Data extraction

Data gathered on general information regarding Neurology, the number of neurologists in Brazil, and first-year Neurology residency positions were measured by headcount and were extracted from the Brazilian Medical Demography research database, concerning the years 2011, 2013, 2015, 2018, and 2020. 9 10 11 12 13 The methodology used in the Brazilian Medical Demography was previously described. 14 Briefly, the Brazilian Medical Demography database included administrative registry and notary data obtained after linkage of three primary sources: the Brazilian Federal Council of Medicine (institution which supervises and regulates medical practice), a database that centralized information from 27 Regional Councils of Medicine, including one database from each Brazilian state; the National Medical Residency Committee's database, affiliated to the Ministry of Education, which aggregates information by those who attended or are currently attending a medical residency program; and the Brazilian Medical Association dataset that integrated databases from medical specialty societies, like the Brazilian Academy of Neurology. We defined as “neurologist” only physicians with complete training in an official Neurology residency program (data from the National Medical Residency Committee database) or approved in direct exams provided by the Brazilian Academy of Neurology, which have legal validity (data from the Brazilian Medical Association dataset). Registrations in more than one state were limited to less than 10% of the neurologists (2015: 8.3; 2018: 9.32; and 2020: 9.6%). 11 12 13

Estimates of newly graduated medical students per state were calculated using data obtained (global number of graduated medical students and proportion of overall graduated students per state) in reports of the Brazilian National Institute for Educational Studies and Research, 14 a special research agency linked to the Ministry of Education, responsible for assessing basic and higher education nationally. Data from the Index of Development of Basic Education – 2019 were also extracted from the Brazilian National Institute for Educational Studies and Research. 15

Population estimates, urbanization rates, and geographical data were extracted from the Brazilian Institute of Geography and Statistics, 16 17 the main provider of data and information about the country. Household income per capita (in Brazilian real) was extracted from the Continuous National Household Sample Survey 2020. 18 Data from the Municipal Human Development Index – 2010 was used. 19 Total gross realized revenues (for 2019, in Brazilian real) were obtained from the National Treasury of Brazil. 20

### Statistical analysis

To evaluate the evolution of neurologists in Brazil (regions and states), we used two measures: demographic distribution (number of neurologists per 100,000 population) and geographic distribution (number of neurologists per 10,000 km ^2^ ). To evaluate the evolution of Neurology residency positions in Brazil (regions and states), we used two measures: the proportion of first-year Neurology residency positions per 1,000,000 population and the proportion of first-year Neurology residency positions per 1,000 newly graduated medical students. We performed the Spearman correlations to analyze the association of the demographic distribution of neurologists with measures of wealth (household income per capita, the Municipal Human Development Index, and total gross realized revenues) between states. We also performed multiple linear regression analyses using the demographic distribution of neurologists as the dependent variable and measures of wealth, education (Index of Development of Basic Education), proportion of population aged 60 years or older, and urbanization rate as independent variables between states. These socioeconomic variables were elected due to their potential to affect the neurologist workforce. Statistical tests were performed using the Statistical Package Social Sciences (SPSS, IBM Corp. Armonk, NY, USA) for Windows, version 23.0, and graphical representations were generated using the R software (R Foundation for Statistical Computing, Vienna, Austria) version 4.0.4 and the R package *ggplot2* .

### Data availability

The datasets generated during the current study are available from the corresponding author on request.

## RESULTS

### Evolution of neurologists in Brazil from 2011 to 2020

The median age of neurologists in Brazil, considering the period mentioned above, was 47.3 years. Less than 6% of neurologists are younger than 30 years old (Neurology residents excluded), and around a quarter of neurologists are older than 60 years. Over the last decade, a growing number of women pursued a neurological career, and they worked an average period of 22 to 25 years ( Table 1 ). Since 2011, the number of neurologists in Brazil has increased in all regions and states. The highest increase in the number of neurologists was in the North region, as well as this was the region with the highest populational increase over this period ( Table 2 ). Roraima and Tocantins had the highest growth rate, while Rio de Janeiro and the Rio Grande do Sul had the lowest growth rate ( Table 2 ).

**Table 1:** Characteristics of neurologists in Brazil, from 2011 to 2020

	2011 (n = 2,629)	2013 (n = 3,212)	2015 (n = 4,362)	2018 (n = 5,104)	2020 (n = 5,779)
**Sex (Female, %)**	36.2	35.46	40.1	41.5	41.9
**Mean time since graduation (years)**	NA	25.15	23.5	22.2	NA
**Mean age (years)**	48.28	49.36	46.8	47.1	47.3
**Age < 30 years old (%)**	NA	4.11	5.9	4.9	5.2
**Age 30–60 years old (%)**	NA	71.2	71.5	73.6	71.5
**Age > 60 years old (%)**	NA	24.69	22.6	21.4	23.3

**Abbreviation:** NA, Not available.

**Table 2:** Number of neurologists and demographic distribution of neurologists (number of neurologists per 100,000 population) in Brazil, from 2011 to 2020

State	Population (2020)	2011		2013		2015		2018		2020		Growth Rate, %		
		n	n_p	n	n_p	n	n_p	n	n_p	n	n_p	neurol	pop_BR	n_p%
Acre	894,470	1	0.13	1	0.13	4	0.5	5	0.58	5	0.56	400	19.84	**317.22**
Amapá	861,773	2	0.29	2	0.27	3	0.39	4	0.48	5	0.58	150	25.93	**98.52**
Amazonas	4,207,714	14	0.40	29	0.76	41	1.04	47	1.15	57	1.35	307.14	18.92	**242.38**
Pará	8,690,745	22	0.29	33	0.41	43	0.53	58	0.68	70	0.81	218.18	13.03	**181.49**
Rondônia	1,796,460	6	0.38	12	0.69	15	0.85	20	1.14	20	1.11	233.33	13.96	**192.51**
Roraima	631,181	1	0.22	2	0.41	4	0.79	4	0.69	6	0.95	500	37.16	**337.43**
Tocantins	1,590,248	3	0.21	6	0.41	9	0.59	13	0.84	16	1.01	433.33	13.52	**369.83**
**North region**	**18,672,591**	**49**	**0.3**	**85**	**0.5**	**119**	**0.68**	**151**	**0.83**	**179**	**0.96**	**265.31**	**16.01**	**214.88**
Alagoas	3,351,543	21	0.67	28	0.85	35	1.05	43	1.29	55	1.64	161.9	6.62	**145.64**
Bahia	14,930,634	97	0.69	124	0.82	161	1.06	188	1.27	221	1.48	127.84	5.91	**115.12**
Ceará	9,187,103	50	0.59	74	0.84	118	1.33	148	1.63	169	1.84	238	7.7	**213.83**
Maranhão	7,114,598	13	0.2	27	0.4	47	0.68	54	0.77	58	0.82	346.15	7.05	**316.75**
Paraíba	4,039,277	25	0.66	33	0.84	46	1.16	55	1.38	67	1.66	168	6.54	**151.55**
Pernambuco	9,616,621	92	1.04	106	1.15	138	1.48	173	1.82	198	2.06	115.22	8.48	**98.3**
Piauí	3,281,480	14	0.45	18	0.57	42	1.31	48	1.47	60	1.83	328.57	4.49	**310.14**
Rio Grande do Norte	3,534,165	19	0.59	24	0.71	45	1.31	56	1.61	64	1.81	236.84	10.49	**204.86**
Sergipe	2,318,822	20	0.96	27	1.23	32	1.43	37	1.62	45	1.94	125	10.96	**102.78**
**Northeast region**	**57,374,243**	**351**	**0.66**	**461**	**0.83**	**664**	**1.17**	**805**	**1.41**	**937**	**1.63**	**166.95**	**7.24**	**148.93**
Espírito Santo	4,064,052	57	1.61	75	1.95	105	2.67	125	3.15	136	3.35	138.6	14.58	**108.24**
Minas Gerais	21,292,666	270	1.37	328	1.59	428	2.05	502	2.39	594	2.79	120	7.93	**103.84**
Rio de Janeiro	17,366,189	324	2.01	363	2.22	471	2.85	535	3.12	577	3.32	78.09	7.78	**65.23**
São Paulo	46,289,333	828	1.99	929	2.13	1,290	2.91	1,563	3.43	1,768	3.82	113.53	11.31	**91.84**
**Southeast region**	**89,012,240**	**1,479**	**1.83**	**1,695**	**2.01**	**2,294**	**2.68**	**2,725**	**3.11**	**3,075**	**3.45**	**107.91**	**9.92**	**89.14**
Paraná	11,516,840	177	1.68	229	2.08	303	2.71	332	2.93	369	3.2	108.47	9.56	**90.29**
Rio Grande do Sul	7,252,502	251	2.34	312	2.79	391	3.48	415	3.66	459	4.02	82.87	6.43	**71.82**
Santa Catarina	11,422,973	111	1.76	139	2.1	192	2.82	218	3.08	238	3.28	114.41	14.81	**86.76**
**South region**	**30,192,315**	**539**	**1.96**	**680**	**2.36**	**886**	**3.03**	**965**	**3.24**	**1,066**	**3.53**	**97.77**	**9.54**	**80.55**
Federal District	3,055,149	83	3.18	123	4.41	174	5.97	188	6.32	212	6.94	155.42	17.06	**118.21**
Goiás	7,113,540	80	1.32	103	1.6	132	2.0	168	2.43	183	2.57	128.75	16.99	**95.54**
Mato Grosso	3,526,220	21	0.68	31	0.97	42	1.29	46	1.34	53	1.5	152.38	14.64	**120.15**
Mato Grosso do Sul	2,809,394	27	1.09	34	1.31	51	1.92	59	2.15	74	2.63	174.07	13.39	**141.7**
**Center-West region**	**16,504,303**	**211**	**1.48**	**291**	**1.94**	**399**	**2.58**	**461**	**2.87**	**522**	**3.16**	**147.39**	**15.87**	**113.52**
**Brazil**	**211,755,692**	**2,629**	**1.37**	**3,212**	**1.6**	**4,362**	**2.13**	**5,104**	**2.45**	**5,779**	**2.73**	**119.82**	**10.07**	**99.7**

Abbreviations: n, number of neurologists; n_p, number of neurologists per 100,000 population; neurol., growth rate of number of neurologists; n_p%, growth rate of number of neurologists per 100,000 population; pop_BR, growth rate of the Brazilian population. **Note:** The growth rate indicates the percentage of the increase in numbers from 2011 to 2020. Data about the number of neurologists in 2012, 2014, 2016, 2017, and 2019 were not available.

**Figure 1:**
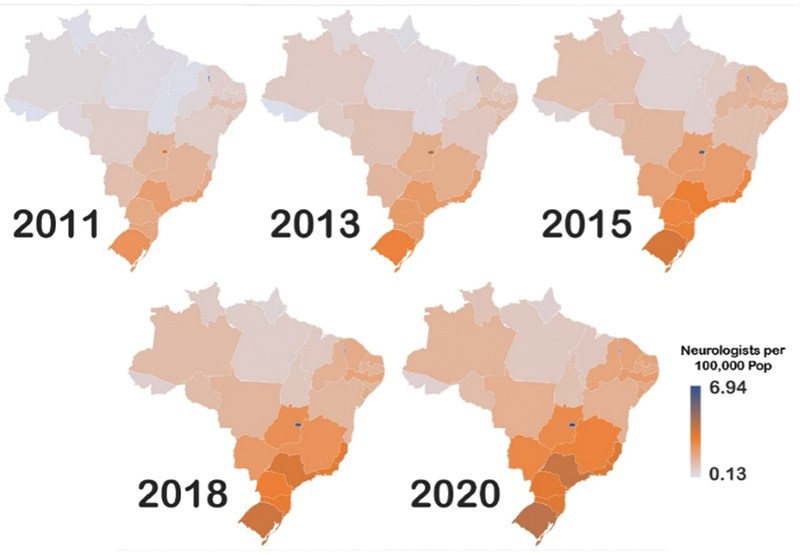
Map chart of the demographic distribution of neurologists in Brazil from 2011 to 2020. Data about the number of neurologists in 2012, 2014, 2016, 2017, and 2019 were not available.

**Figure 2:**
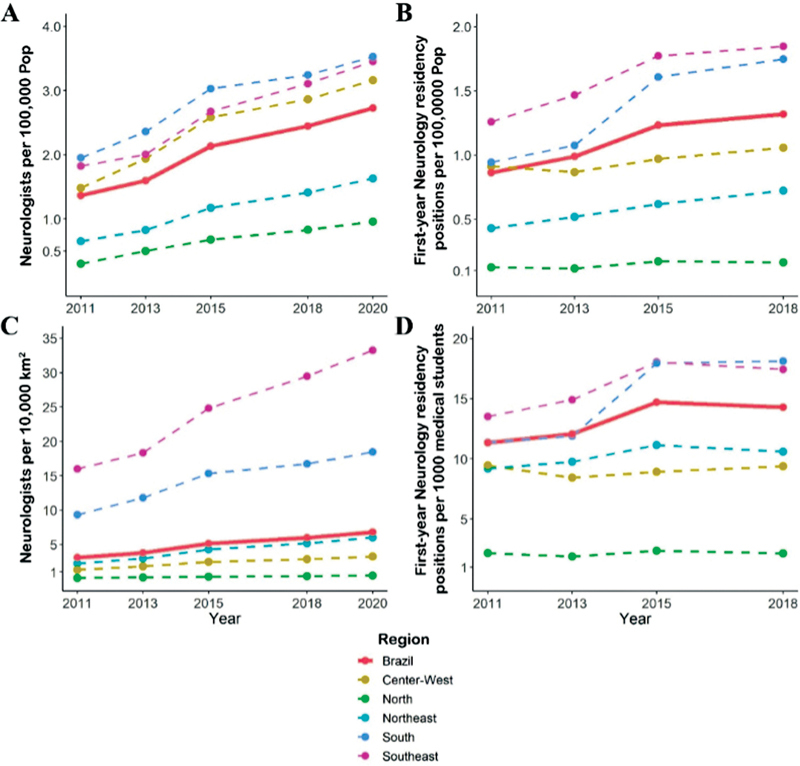
Distribution of neurologists and first-year Neurology residency positions in Brazil from 2011 to 2020. A: Demographic distribution of neurologists. B: First-year Neurology residency positions per 100,000 population. C: Geographic distribution of neurologists. D: First-year Neurology residency positions per 1,000 newly graduated medical students. Data about the number of neurologists in 2012, 2014, 2016, 2017, and 2019, as well as Neurology residency positions in 2020 were not available.

The number of neurologists per 100,000 population in Brazil increased by two. Regarding demographic distribution, the Northern region had the highest increase in the proportion of neurologists. There are remarkable and longstanding geographical inequalities in the distribution of neurologists between North (lowest proportion) and South (highest proportion). Moreover, the Northeastern region, which also had a proportion of neurologists per 100,000 population lower than 1 in 2011, achieved an increase in 2020 ( Figure 1 and 2A ). In 2020, the Federal District and Rio Grande do Sul had the highest proportion of neurologists per 100,000 population, while Acre and Amapá had the lowest ( Table 2 ).

There was a stability in the number of neurologists per 10,000 km ^2^ in the North, Center-West, and South, and an increase in the Northeast and Southeast ( Figure 2C ). Similar to demographic distribution, the Federal District and the Rio Grande do Sul had the highest proportions of neurologists per 10,000 km ^2^ in 2020, and Roraima and Acre had the lowest proportions ( Table 3 ).

**Table 3:** Geographic distribution of neurologists in Brazil (number of neurologists per 10,000 km ^2^ ), from 2011 to 2020

State	% Forest Area	2011	2013	2015	2018	2020	Growth Rate, %
Acre	63.10	0.06	0.06	0.24	0.3	0.3	400
Amapá	85.14	0.14	0.14	0.21	0.28	0.35	150
Amazonas	87.37	0.09	0.19	0.26	0.3	0.37	307.14
Pará	65.94	0.18	0.26	0.35	0.47	0.56	218.18
Rondônia	53.68	0.25	0.50	0.63	0.84	0.84	233.33
Roraima	81.81	0.04	0.09	0.18	0.18	0.27	500
Tocantins	19.37	0.11	0.22	0.32	0.47	0.58	433.33
**North Region**	**72.02**	**0.13**	**0.22**	**0.31**	**0.39**	**0.46**	**265.31**
Alagoas	2.06	7.55	10.06	12.58	15.45	19.76	161.9
Bahia	2.66	1.72	2.2	2.85	3.33	3.91	127.84
Ceará	0.91	3.36	4.97	7.93	9.94	11.35	238
Maranhão	13.29	0.39	0.82	1.43	1.64	1.76	346.15
Paraíba	0.73	4.43	5.84	8.15	9.74	11.87	168
Pernambuco	4.52	9.38	10.81	14.07	17.64	20.19	115.22
Piauí	6.53	0.56	0.71	1.67	1.91	2.38	328.57
Rio Grande do Norte	0.45	3.6	4.54	8.52	10.6	12.12	236.84
Sergipe	1.69	9.12	12.31	14.59	16.87	20.51	125
**Northeast Region**	**5.33**	**2.26**	**2.97**	**4.28**	**5.17**	**6.04**	**166.95**
Espírito Santo	2.17	12.37	16.28	22.79	27.13	29.52	138.6
Minas Gerais	2.55	4.60	5.59	7.3	8.56	10.13	120
Rio de Janeiro	8.78	74.06	82.97	107.66	122.28	131.88	78.09
São Paulo	4.56	33.36	37.43	51.97	62.97	71.23	113.53
**Southeast Region**	**3.37**	**16.0**	**18.33**	**24.81**	**29.47**	**33.26**	**107.91**
Paraná	3.13	8.88	11.49	15.2	16.66	18.51	108.47
Rio Grande do Sul	1.32	8.91	11.08	13.88	14.73	16.29	82.87
Santa Catarina	3.74	11.6	14.52	20.06	22.77	24.86	114.41
**South Region**	**2.35**	**9.35**	**11.79**	**15.36**	**16.73**	**18.48**	**97.77**
Federal District	14.29	144.08	213.51	302.04	326.34	368.01	155.42
Goiás	2.57	2.35	3.03	3.88	4.94	5.38	128.75
Mato Grosso	21.47	0.23	0.34	0.47	0.51	0.59	152.38
Mato Grosso do Sul	3.44	0.76	0.95	1.43	1.65	2.07	174.07
**Center-West Region**	**13.43**	**1.31**	**1.81**	**2.48**	**2.87**	**3.25**	**147.39**
**Brazil**	**36.62**	**3.09**	**3.77**	**5.13**	**6.0**	**6.79**	**119.82**

**Notes:** The growth rate indicates the percentage of the increase in numbers from 2011 to 2020. Data about the number of neurologists in 2012, 2014, 2016, 2017, and 2019 were not available. Aiming to estimate the amount of non-urban areas in Brazil with low population density, we calculated the percentage of forest area (% Forest Area) by state with data extracted from the National Registry of Public Forests 2017, which includes federal, state, and municipal public forests.

### Evolution of neurology residency positions in Brazil from 2010 to 2019

In 2011, there were 157 first-year Neurology residency positions in Brazil, and 13 states had no Neurology training programs. In 2019, the number of positions offered increased to 299. Out of the 13 states mentioned above, 8 still did not have Neurology residency programs available, 5 of which are from the Northern region (Acre, Amapá, Rondônia, Roraima, and Tocantins) ( Table 4 ).

**Table 4:** First-year positions in Neurology residency programs in Brazil, from 2010 to 2019

State	2010	2011	2012	2013	2014	2015	2016	2017	2018	2019	Growth Rate, %
Acre	0	0	0	0	0	0	0	0	0	0	NA
Amapá	0	0	0	0	0	0	0	0	0	0	NA
Amazonas	2	2	2	2	2	2	2	3	1	2	0.0
Pará	0	0	0	0	1	1	1	1	2	2	NA
Rondônia	0	0	0	0	0	0	0	0	0	0	NA
Roraima	0	0	0	0	0	0	0	0	0	0	NA
Tocantins	0	0	0	0	0	0	0	0	0	0	NA
**North Region**	**2**	**2**	**2**	**2**	**3**	**3**	**3**	**4**	**3**	**4**	**100**
Alagoas	0	0	0	0	0	0	1	1	1	2	NA
Bahia	6	6	8	8	11	11	12	13	13	13	116.67
Ceará	7	7	8	8	10	10	10	10	10	13	85.71
Maranhão	0	0	0	0	0	0	0	0	0	0	NA
Paraíba	0	0	0	0	0	0	0	0	0	0	NA
Pernambuco	9	9	12	9	10	10	12	13	14	13	44.44
Piauí	0	0	0	0	0	0	0	0	0	2	NA
Rio Grande do Norte	1	1	1	2	2	2	2	3	1	2	100
Sergipe	0	0	2	2	2	2	2	2	2	2	NA
**Northeast Region**	**23**	**23**	**31**	**29**	**35**	**35**	**39**	**42**	**41**	**47**	**104.35**
Espírito Santo	1	0	0	0	0	0	0	0	0	0	NA
Minas Gerais	16	16	19	25	23	36	35	40	39	38	137.5
Rio de Janeiro	22	24	23	30	23	22	27	23	23	23	4.55
São Paulo	55	62	63	69	91	94	94	96	100	109	98.18
**Southeast Region**	**94**	**102**	**105**	**124**	**137**	**152**	**156**	**159**	**162**	**170**	**80.85**
Paraná	9	9	10	11	16	16	20	22	21	22	144.44
Rio Grande do Sul	9	10	13	13	18	21	21	22	21	25	177.78
Santa Catarina	7	7	7	7	10	10	11	10	10	11	57.14
**South Region**	**25**	**26**	**30**	**31**	**44**	**47**	**52**	**54**	**52**	**58**	**132**
Distrito Federal	6	6	6	5	5	6	6	6	6	7	16.67
Goiás	7	7	7	7	8	8	8	8	8	10	42.86
Mato Grosso do Sul	0	0	0	0	0	0	0	2	2	2	NA
Mato Grosso	0	0	0	1	1	1	1	1	1	1	NA
**Center-West Region**	**13**	**13**	**13**	**13**	**14**	**15**	**15**	**17**	**17**	**20**	**53.85**
**Brazil**	**157**	**166**	**181**	**199**	**233**	**252**	**265**	**276**	**275**	**299**	**90.45**

**Abbreviation:** NA, not available. **Notes:** Data about Neurology residency positions in 2020 were not available. Growth rate indicates the percentage of the increase in numbers from 2010 to 2019, and growth rates for states with no Neurology residency positions in 2010 were not calculated.

The proportions of first-year Neurology residency training per 1,000,000 population between regions persisted stable since 2010. Again, the North had the lowest proportion ( Figure 2B ). Adjusting the number of first-year Neurology residency positions for the estimated number of newly graduated medical students, the South and Southeast provided approximately 18 first-year Neurology residency positions per 1,000 graduated medical students in 2018, higher than the nationwide proportion. The North provided only two positions per 1,000 graduated medical students in 2018 ( Figure 2D ).

### Association of the number of neurologists in Brazil with social inequality

**Figure 3:**
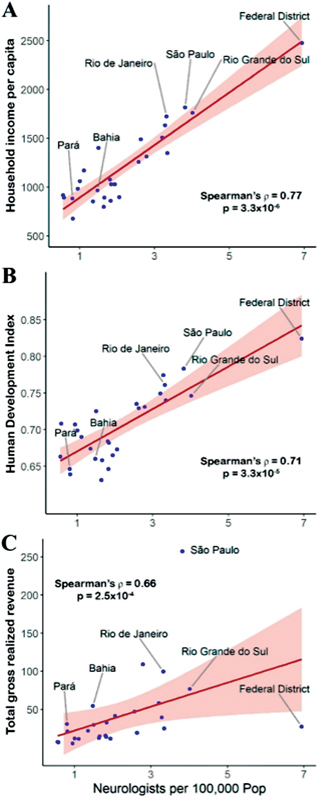
Correlations between the number of neurologists per 100,000 population of Brazilian states in 2020 with household income per capita in 2020 (A), the Municipal Human Development Index in 2010 (B), and total gross realized revenues in 2019 (C).

Correlations showed that states with a lower number of neurologists per 100,000 population had worse indicators of wealth ( Figure 3 ). Also, states with higher growth rates of neurologists had the worse wealth indicators (Municipal Human Development Index: Spearman rho = 0.62, *p* = 0.001).

We performed multiple regression analyses with indicators of wealth, education, the proportion of population aged 60 years or older, and urbanization rate. The results showed that a model with household income, the Index of Development of Basic Education, and the Municipal Human Development Index as independent variables was significantly associated with the number of neurologists per 100,000 population, with an adjusted R ^2^ = 0.845 (F _2,24_ = 71.6, *p* < 0.001). Both household income (β = 1.19, *p* < 0.01) and the Index of Development of Basic Education (β = 0.24, *p* = 0.01) had a positive effect on the demographic density of neurologists, and the Municipal Human Development Index (β = -0.43, *p* = 0.04) had an inverse effect. There was no multicollinearity between independent variables.

## DISCUSSION

The distribution of neurologists in Brazil was analyzed, and differences were noted considering geographical and demographic aspects from different regions. In the last decade, the absolute number of neurologists in Brazil more than doubled, in a growth rate ten times faster than the overall population growth. Although, it was not effective in reducing the imbalance of neurologists' availability across the country, as most of them remain in the country's wealthiest regions. As the demographic distribution of neurologists has an ecological association with social inequality, the local socioeconomic status may directly influence the uneven distribution of neurologists observed between regions and states.

Brazil has continental dimensions, and population concentration varies widely within different areas. The inequalities in the distribution of neurologists reflected a North-South gradient in wealth indicators.

There are some issues to be addressed, such as the lack of specialized professionals, the insufficient number of neurologists being trained, and the high concentration of neurologists in some specific areas. Some problems like Neurophobia and burnout are not exclusive to Brazil and have also been observed in some developed countries. We previously showed that Neurophobia was present in Brazilian medical students, suggesting the fear of Neurology may affect the number of future neurologists. 21 As in many developed countries, burnout syndrome is also common among neurologists in Brazil, leading to early retirement. 22 Finally, state capitals in Brazil have high concentrations of physicians compared to other cities. 13 Urban areas also present a high number of physicians than the countryside, 13 showing that neurologists in Brazil tend to work in the urban and wealthiest areas.

Over the last ten years, the number of offered first-year Neurology positions increased. However, admission to these programs remains limited for new doctors due to different issues. First, teaching facilities are not evenly distributed along with different states in Brazil. Second, the number of offered positions is insufficient, mainly in the areas with the highest demand for specialists. Undeniably, training new neurologists for clinical practice is a considerable challenge, observed in all specialist training programs.

In the developing world, most Arab 23 and African countries, 24 as well as India, 25 have an insufficient supply of neurologists. Some countries have no neurologists at all, leading to a substantial worsening on neurological care. At the opposite extreme, upper-middle and high-income countries have the highest proportions of neurologists per population (1 neurologist per 10,000–50,000 population), 26 notwithstanding that these rich countries also have issues with their neurological supply, and many complain of a shortage of neurologists. 5 According to this study, Brazil has a ratio of 2.73 neurologists per 100,000 population. Previous reports described the following values for countries comparable to Brazil: Russia, 5.43; USA, 5.2; Australia, 2; Mexico, 0.33; South Africa, 0.25; and India, 0.08. 5 27

The perception of lack of neurologists is real worldwide. The possible causes are diverse, as previously mentioned, such as Neurophobia, high rates of burnout, the concentration of workforce in large cities, search for better wages. 5 The scenario is driving the national societies of Neurology to stimulate medical students to choose Neurology as a future career: the American Academy of Neurology is investing in a change of image of neurologists, emphasizing intervention, prevention, and rehabilitation, and the Japanese Society of Neurology combines marketing and a costly program of immersion training in Neurology for medical students to increase their numbers. 5

Overall, the proportion of neurologists per 100,000 population in Brazil is up to three times the suggested number by WHO (1:100,000), and even the most neurologist-scarce region in Brazil, the North, meets the proposed goal. However, WHO established this target proportion 24 years ago and, since then, the burden of all neurological diseases worldwide has increased, accompanying the increase of the world population and its aging. 1 It is reasonable to consider that this proportion of neurologists per population should be revised and no longer meets global needs. Our data suggests Brazil is facing similar problems as some developed countries regarding Neurology assistance.

Apart from that, Brazil has additional difficulties in dealing with the low number of programs qualified to train new neurologists and consequently a low number of Neurology residency positions. The deficiency has been more dramatic in areas with high demand for neurological care. Medical residency is the most effective program to guarantee proper training for young neurologists in Brazil. As there are few institutions that can offer high-quality assistance and education, the current scenario has a negative effect on neurological healthcare.

Medical students need to feel inspired to pursue Neurology as their future career, but it would be fruitless if there were no training centers. Moreover, without a substantial increase in Neurology residency positions, the neurological workforce will fail to improve its assistance in all parts of Brazil. There is a need to support small and medium Neurology services in these states, providing them with more resources to create new Neurology residency positions. The Brazilian Academy of Neurology should be part of this effort. The establishment of a critical mass of neurologists in strategic rural cities might lead to the empowerment of existing and new Neurology residency programs and the generation of new positions.

Unsurprisingly, there was an ecological association between the demographic distribution of neurologists in Brazil with general socioeconomic conditions. Wealthy regions of rich countries have high numbers of neurologists, while rural areas in emerging countries have no neurologists. Economic expansion is a major determinant of medical assistance, particularly for medical specialties such as Neurology. 28 Furthermore, the distribution of physicians in the public and private sectors may be associated with inequality. In Brazil, the regions with a lesser proportion of physicians working exclusively on the private sector (North and Northeast) had lesser demographic densities of neurologists. 13

Better working conditions and higher wages may help establish neurologists outside more prominent cities, as well as the existence of an infrastructure dedicated to neurological care (neurological beds, sub-specialized neurological services, rehabilitation clinics) in strategic cities in rural areas. Policies supporting the establishment of a critical mass of neurologists in rural areas, who could work together, share responsibilities, provide continuing medical education, and serve as faculty to train more specialists could help neurologists settle in the countryside.

Brazilian Neurology has great challenges to deal with during the upcoming years. It would be wise if neurologists from the National Health System and Universities, public health authorities, primary care physicians, and patients sat together to study the data and propose solutions. Recently, a strategic plan was proposed by the American Academy of Neurology to deal with the shortage of neurologists in the USA, 29 which can be used as a model for Brazil.

The main strength of our study relies on the linkage of robust databases containing documented information about specialists in Brazil. Even though the information was initially collected for administrative purposes, data analysis can lead to thoughtful insights. Limitations related to the use of secondary data must be taken into account. Using simple headcount instead of full-time equivalent to estimate the supply of services does not consider physicians' productivity, working hours per week, or the scope of delivered services. Headcount data have been associated with the overestimation of physician-to-population ratios. Physician-to-population ratios may not represent the need for neurological care in distinct geographic regions. 30 Thus, a utilization-based approach for future planning of the Brazilian neurologist workforce may be more beneficial than targeting the 1 to 100,000 magic ratio.

In conclusion, Brazil advanced in providing neurologists enough to meet the demands of the population; however, the inequality in their distribution through regions still impairs the quality of neurological care in more impoverished areas. The training of new neurologists is also marked by inequality between regions, with a bottleneck allowing few graduated medical students to become neurologists. Neurologists, public health authorities, and patients should discuss solutions to deal with this inequality.
